# Transplacental transfer of maternal SARS-CoV-2 antibodies in dichorionic and monochorionic twin pregnancies

**DOI:** 10.1371/journal.pone.0328137

**Published:** 2025-09-02

**Authors:** Jennifer E. Stolarczuk, Monica Sosa, Mindy Pike, Alexis Baranoff, Ava Lekander, Hye Cho, Erin A. Goecker, Alexander L. Greninger, Linda O. Eckert, Janet A. Englund, Alisa Kachikis

**Affiliations:** 1 University of Washington School of Medicine, Seattle, Washington, United States of America; 2 Department of Obstetrics and Gynecology, University of Washington, Seattle, Washington, United States of America; 3 SUNY Upstate Medical University, Syracuse, New York, United States of America; 4 Department of Laboratory Medicine and Pathology, University of Washington, Seattle, Washington, United States of America; 5 Department of Global Health, University of Washington, Seattle, Washington, United States of America; 6 Department of Pediatrics, University of Washington, Seattle, Washington, United States of America; 7 Seattle Children’s Research Institute, Seattle, Washington, United States of America; Kasr Alainy Medical School, Cairo University, EGYPT

## Abstract

**Background:**

Maternal immunization relies on active transplacental transfer of immunoglobulin G. However, very little is known about the efficacy of maternal immunization in the setting of multiple gestation. We aimed to investigate transplacental transfer of maternal antibody in pregnancies with multiple gestation including mono- and dichorionic pregnancies by evaluating anti-Spike antibody transfer at delivery.

**Methods:**

We conducted a cohort study among individuals with singleton or twin pregnancies who received ≥ 2 doses of an mRNA COVID-19 vaccine before delivery. We tested paired maternal and cord blood samples for anti-Spike antibody levels via Roche Elecsys® immunoassays and used linear regression to evaluate associations between pregnancy type and anti-Spike antibody levels. We included as covariates gestational age at birth, timing of last vaccine dose, number of vaccine doses, and small for gestational age birth weight.

**Results:**

Between 2021−2023, we tested paired maternal and cord samples for anti-Spike antibody from 362 singleton pregnancies and 36 twin pregnancies, of which 12 and 24 were monochorionic and dichorionic, respectively. After adjusting for covariates, maternal and cord anti-Spike antibody concentrations were significantly lower in twin pregnancies compared to singleton pregnancies (beta:-0.91, 95% Confidence Interval [CI]: −1.62,-0.19; p = 0.01. beta −1.20, 95% CI: −2.36,-0.04; p = 0.04), however there was no difference in cord:maternal antibody ratios. After adjusting for covariates, there was no difference in maternal and cord antibody concentrations between dichorionic and monochorionic pregnancies, however, cord:maternal antibody ratios remained significantly lower in monochorionic compared to dichorionic pregnancies (beta:-0.49; 95% CI: −0.86,-0.13; p = 0.01).

**Conclusions:**

Twin and singleton infants have similar cord:maternal maternally derived SARS-CoV-2 antibody ratios after maternal COVID-19 vaccine although maternal and cord antibody concentrations are lower in infants from twin pregnancies and specifically antibody transfer is less efficient in monochorionic pregnancies. Further research is needed to investigate impaired transplacental IgG transfer in high-risk pregnancies.

## Introduction

The immunization of pregnant persons against infections offers dual benefit to both pregnant individuals and their fetuses and neonates. Maternal immunization describes the administration of vaccinations during pregnancy to boost maternal antibody concentrations and enhance active maternal-fetal transfer of vaccine-specific maternal Immunoglobulin G (IgG) [1,2]. Maternal IgG binds to neonatal fragment crystallizable receptors (FcRn) on the syncytiotrophoblast of chorionic villi [3]. The maternal IgG is released into fetal capillaries and helps provide immune protection for neonates and infants in the first months of life. Vaccination in both uncomplicated and complicated pregnancies is a key strategy to prevent or mitigate severe illness from infectious disease, particularly respiratory tract infections, in pregnant persons and their infants [4]. For example, administration of COVID-19 vaccines before or during pregnancy has been shown to protect pregnant individuals and their infants against severe infection and hospitalization from SARS-CoV-2 [5–7]. However, there is little research on the efficiency of transplacental transfer of SARS-CoV-2-specific antibodies in twin pregnancies.

Multiple factors may affect transplacental antibody transfer including gestational age of the pregnancy, total IgG antibody, IgG antibody subclass, properties of the antigen, maternal co-morbidities and placental factors [2]. Physiologic adaptations of pregnancy are often magnified in twin compared to singleton pregnancies, which may impact placental function and maternal vaccination [8]. For example, it is plausible that the further amplified increases in maternal blood volume and hemodilution in a twin pregnancy may result in lower maternal IgG concentration after vaccination, or impaired transplacental IgG transfer. In addition, infants from twin pregnancies are particularly at risk for prematurity and low birth weight compared to infants from singleton pregnancies [9–11]. These risks are further increased in monochorionic compared to dichorionic twin pregnancies [12–14]. Due to a vulnerable immune system and small airways, prematurity places infants at increased risk of infection by respiratory pathogens such as SARS-CoV-2; thus, infants rely on transplacental transfer of maternally derived IgG prior to birth as a key protection mechanism during the first few months of life [4].

Few studies have assessed transplacental antibody transfer in twin pregnancies compared to singleton pregnancies. One study of 50 twin pregnancies found that the mean maternal IgG level was higher in the umbilical cord than the maternal serum; and the cord IgG levels were in range for singletons of the same gestational age [15]. Stach et al. showed that umbilical cord total IgG concentrations were higher in dichorionic compared to monochorionic twin pregnancies [16]. However, the impact of twin pregnancy compared to singleton pregnancy and of chorionicity within twin pregnancies on transplacental transfer of SARS-CoV-2 IgG has not been studied. Our study seeks to understand how multiple gestation and chorionicity impacts transplacental maternal IgG transfer by investigating COVID-19 vaccines and SARS-CoV-2 anti-Spike (S) antibody transfer in monochorionic and dichorionic twin and singleton pregnancies.

## Materials and methods

### Participants

We recruited participants and acquired informed written consent for an ongoing prospective cohort study on maternal immunizations at the University of Washington (UW). Maternal blood samples were collected within 72 hours of delivery and cord blood samples at delivery. We included samples from participants with a singleton and monochorionic or dichorionic twin pregnancies. All subjects received two or more doses of an mRNA based COVID-19 vaccine prior to delivery, had no known significant fetal genetic condition or structural anomaly, and paired maternal and cord blood samples as well as complete SARS-CoV-2-specific antibody data were available. Samples were excluded if participant received less than two doses of a COVID-19 vaccine prior to delivery, received a non-mRNA-based vaccine, had a triplet pregnancy or had incomplete SARS-CoV-2-specific antibody data. Ethics approval for the study was received by the UW Human Subjects Division.

### Variables

We collected clinical data by performing chart abstraction from electronic medical records (EMR) and organized data into our database through REDCap (version 13.10.6, 2024) as previously described [17]. In short, we categorized participant race and ethnicity data using the Centers for Disease Control and Prevention’s categories [18]. Participant insurance status was categorized by public, private or Tricare/Military, Federal, or other. We calculated body mass index (BMI) using maternal weight at delivery. We used the American College of Obstetricians & Gynecologists’ (ACOG) criteria to define pregestational diabetes, preeclampsia with and without severe features, chronic hypertension, chronic hypertension with superimposed preeclampsia, and eclampsia [19]. Designated autoimmune diseases/inflammatory disorders included conditions such as systemic lupus erythematous and Crohn’s disease, and rheumatoid arthritis, among others. Patients on long-term corticosteroids, biologics, or other immunosuppressive medications were categorized as receiving immunosuppressing medications. Participants’ number of COVID-19 doses vaccine data was collected through the Washington State Immunization Registry data linked to the EMR. COVID-19 infection status prior to or during pregnancy was determined through provider indication or positive test result. In addition, twin pregnancy chorionicity was determined via ultrasound report in the EMR.

### Antibody testing

We tested maternal and cord blood samples for anti-S and anti-Nucleocapsid (N) antibody concentrations using the semi-quantitative Roche Elecsys® immunoassays at the University of Washington Virology Laboratory which has a reported sensitivity of 99.5% and specificity of 99.8% for SARS-CoV-2 anti-N detection [20]. In our analysis, we converted results from the Roche Elecsys® Anti-SARS-CoV-2 S immunoassay to binding antibody units (BAU)/mL and log_2_-transformed antibody levels. We defined evidence of past SARS-CoV-2 infection to be positive COVID-19 antigen or a PCR test, or positive anti-N serology. Maternal total IgG (mg/dL) was tested in the UW Immunology Clinical Laboratory using an Optilite analyzer with standard reagents.

### Analysis

We reported demographic, pregnancy, and neonatal characteristics as absolute numbers with percentages or as medians with interquartile ranges (IQR). We compared characteristics using t-tests, Wilcoxon rank-sum tests, chi-square tests, and Fisher’s exact tests. We analyzed relationships between type of pregnancy (singleton versus twin) as well as chorionicity (monochorionic versus dichorionic) and maternal and cord anti-S antibody levels using Wilcoxon rank sum tests and linear regression analyses. Similar analyses were performed for the ratio of cord to maternal (cord:maternal) anti-S antibody concentrations based on untransformed values, which were tested with t-tests and linear regression. Cord:maternal antibody ratios of >1 are generally considered “efficient” placental antibody transfer in published studies [1], however are distinct and may not correlate to correlates of protection against a specific antigen. Anti-S IgG antibody levels were log2-transformed and reported as geometric mean concentrations (GMC) and 95% confidence intervals (CI). A boxplot of cord:maternal ratios for singleton, monochorionic, and dichorionic pregnancies was created using the median, interquartile range, and range of the ratio in each group to show differences. We selected covariates a priori and based on significant associations with the exposure of twin gestation or chorionicity and outcomes of maternal and cord anti-S antibody concentrations. Covariate associations were tested using chi-square tests, t-tests, and linear regression. For the comparison of singleton versus twin birth, minimally adjusted models included gestational age at delivery and time between last COVID-19 vaccine dose and delivery (in weeks). A fully adjusted model additionally included number of COVID-19 vaccine doses prior to delivery and small for gestational age. For the comparison of monochorionic versus dichorionic, the minimally adjusted linear regression model included time between last COVID-19 vaccine dose and delivery (in weeks). The fully adjusted model additionally included gestational age at delivery and number of vaccine doses before delivery. Linear regression models for cord anti-S antibody levels and cord:maternal antibody ratios allowed for intragroup correlation by specifying clusters of twins, relaxing the requirement that the observations be independent. We performed statistical analyses using Stata version 18.0 (StataCorp, College Station, TX, USA) [21]. We considered a two-sided P-value less than 0.05 as statistically significant, and followed the Strengthening the Reporting of Observational Studies in Epidemiology (STROBE) reporting guideline [22].

## Results

### Baseline demographic and medical characteristics

From February 2021 to April 2023, a total of 398 pregnant participants met the inclusion criteria. Of these, 362 were singleton pregnancies and 36 were twin pregnancies, of which 12 and 24 were monochorionic and dichorionic, respectively. Baseline demographic and medical characteristics are presented in Table 1. There was no difference between groups in any of these recorded variables, including race, ethnicity, insurance status, delivery BMI and rates of pregestational diabetes mellitus, pre-eclampsia, autoimmune/inflammatory disorders, or use of immunosuppressing medication. However, singleton pregnancies had higher rates of chronic hypertension than twin pregnancies (p = 0.04). Maternal total IgG concentrations were greater in singleton pregnancies than in twin pregnancies (p = 0.003), with a median of 692 (IQR 577,821) mg/dL and 525 (392,699) mg/dL, respectively. However, no difference in maternal total IgG levels was noted between mono- and dichorionic pregnancies.

**Table 1:** Demographic and baseline characteristics.

	Total (n = 398)	Twin total (n = 36)				Singleton (n = 362)	p-value**
			Mono (n = 12)	Di (n = 24)	p-value*		
Year of recruitment					0.89		0.01
2021	190 (47.7)	9 (25.0)	3 (25.0)	6 (25.0)		181 (50.0)	
2022	148 (37.2)	19 (52.8)	7 (58.3)	12 (50.0)		129 (35.6)	
2023	60 (15.1)	8 (22.2)	2 (16.7)	6 (25.0)		52 (14.4)	
Maternal age	34 (31, 37)	33 (30, 37)	32 (30, 36)	33 (30, 37)	0.78	34 (31, 37)	0.21
Gravidity	2 (1, 3)	2 (1, 2)	2 (2, 3)	2 (1, 2)	0.14	2 (1, 3)	0.82
Parity	0 (0, 1)	0 (0, 1)	1 (0, 1)	0 (0, 1)	0.47	0 (0, 1)	0.38
Race					1.0		0.15
American Indian or Alaska Native	4 (1.1)	2 (5.7)	0 (0.0)	2 (8.3)		2 (0.6)	
Pacific Islander	1 (0.3)	0 (0.0)	0 (0.0)	0 (0.0)		1 (0.3)	
Asian	62 (16.3)	5 (14.3)	2 (18.2)	3 (12.5)		57 (16.5)	
Black or African American	17 (4.5)	2 (5.7)	0 (0.0)	2 (8.3)		15 (4.4)	
White	296 (77.9)	26 (74.3)	9 (81.8)	17 (70.8)		270 (78.3)	
Hispanic ethnicity	37 (9.5)	3 (8.3)	2 (16.7)	1 (4.2)	0.25	34 (9.6)	1.0
Insurance status					0.44		0.08
Public	48 (12.1)	9 (25.0)	4 (33.3)	5 (20.8)		39 (10.8)	
Private	345 (86.9)	27 (75.0)	8 (66.7)	19 (79.2)		318 (88.1)	
Tricare/ Federal/ Other	4 (1.0)	0 (0.0)	0 (0.0)	0 (0.0)		4 (1.1)	
BMI	30.0 (27.1, 34.6)	31.4 (28.5, 36.0)	29.7 (28.9, 32.6)	32.8 (28.1, 36.7)	0.24	30.0 (27.1, 34.5)	0.10
Pregestational DM	12 (3.0)	1 (2.9)	0 (0.0)	1 (4.4)	1.0	11 (3.1)	1.0
Preeclampsia	68 (17.1)	8 (22.2)	1 (8.3)	7 (29.2)	0.22	60 (16.6)	0.36
Chronic HTN	39 (9.8)	0 (0.0)	0 (0.0)	0 (0.0)	NA	39 (10.8)	0.04
Autoimmune/inflammatory disorder	33 (8.3)	3 (8.3)	1 (8.3)	2 (8.3)	1.0	30 (8.3)	1.0
Immunosuppressing meds	24 (6.0)	2 (5.6)	0 (0.0)	2 (8.3)	0.54	22 (6.1)	1.0
COVID-19 vaccine # before delivery					0.47		0.40
2	178 (44.7)	13 (36.1)	4 (33.3)	9 (37.5)		165 (45.6)	
3	154 (38.7)	15 (41.7)	7 (58.3)	8 (33.3)		139 (38.4)	
4+	66 (16.6)	8 (22.2)	1 (8.3)	7 (29.2)		58 (16.0)	
COVID-19 vaccine # in pregnancy					0.50		0.06
0	71 (17.8)	10 (27.8)	5 (41.7)	5 (20.8)		61 (16.9)	
1	137 (34.4)	15 (41.7)	5 (41.7)	10 (41.7)		122 (33.7)	
2	159 (39.9)	7 (19.4)	1 (8.3)	6 (25.0)		152 (42.0)	
3+	31 (7.8)	4 (11.1)	1 (8.3)	3 (12.5)		27 (7.4)	
Last vaccine to delivery (weeks)	21 (9, 41)	25 (10, 50)	25 (3, 50)	25 (14, 50)	0.65	21 (9, 41)	0.43
GA at last vaccine dose	24 (17, 32)	22 (17, 30)	29 (25, 35)	18 (13, 30)	0.01	24 (17, 32)	0.82
COVID-19 infection before or during pregnancy	67 (18.7)	6 (18.2)	3 (25.0)	3 (14.3)	0.64	61 (18.8)	1.0
Positive serology for anti-N	100 (25.2)	13 (36.1)	3 (25.0)	10 (41.7)	0.47	87 (24.1)	0.16
Maternal total IgG (mg/dL)	688 (565, 818)	525 (392, 699)	410 (335, 634)	578 (464, 731)	0.45	692 (577, 821)	0.003

Notes: median (interquartile range) or N (%); continuous variables compared using t-tests and Wilcoxon rank sum tests and categorical variables compared using Fisher’s exact tests; gestational age at last dose only available in individuals with COVID-19 vaccine during pregnancy

Abbreviations: BMI = body mass index; DM = diabetes mellitus; HTN = hypertension; GA = gestational age; IgG = Immunoglobulin G; anti-N = anti-Nucleocapsid antibody

*p-value denotes significance between monochorionic versus dichorionic pregnancies

**p-value denotes significance between twin versus singleton pregnancies

### Pregnancy outcomes

Pregnancy outcome data is presented in Table 2. Twin pregnancies delivered at an earlier median gestation of 36.0 weeks (IQR 34.1, 36.9) than singleton pregnancies (39.0 weeks, IQR 36.7, 39.9; p < 0.001) with no difference in gestational age at delivery between mono- and dichorionic twins (35.4 weeks, IQR 34.5, 36.4, versus 36.1 weeks, IQR 33.8, 36.9, respectively; p = 0.31). Twin pregnancies had a lower median birth weight (2353 grams, IQR 2034, 2691) than singleton pregnancies (3175 grams, IQR 2648, 3565; p < 0.001) and were more likely to have a neonate admitted to the neonatal intensive care unit (NICU) (twin n = 47, 65.3%; singleton n = 83, 22.9%; p < 0.001).

**Table 2:** Pregnancy outcomes of pregnant persons and their infants included in this study.

	Total (n = 434)	Twin total (n = 72)				Singleton (n = 362)	p-value**
			Mono (n = 24)	Di (n = 48)	p-value*		
Delivery gestational age	38.6 (36.4, 39.7)	36.0 (34.1, 36.9)	35.4 (34.5, 36.4)	36.1 (33.8, 36.9)	0.31	39.0 (36.7, 39.9)	<0.001
Range	23.6-42.0	28.6-38.0	28.6-37.3	32.1-38.0		23.6-42.0	
Delivery <37 weeks	155 (35.7)	58 (80.6)	22 (91.7)	36 (75.0)	0.12	97 (26.8)	<0.001
Delivery ≥37 weeks	279 (64.3)	14 (19.4)	2 (8.3)	12 (25.0)		265 (73.2)	
Mode of delivery					1.0		<0.001
Vaginal delivery	211 (48.6)	20 (27.8)	6 (25.0)	14 (29.2)		191 (52.8)	
Cesarean section	223 (51.4)	52 (72.2)	18 (75.0)	34 (70.8)		171 (47.2)	
Birth weight	3027 (2465, 3485)	2353 (2034, 2691)	2228 (1843, 2520)	2384 (2151, 2738)	0.02	3175 (2648, 3565)	<0.001
Fetal growth restriction	55 (12.7)	9 (12.5)	4 (16.7)	5 (10.4)	0.47	46 (12.7)	0.96
Sex					0.61		0.06
Female	222 (51.2)	44 (61.1)	16 (66.7)	28 (58.3)		178 (49.2)	
Male	212 (48.6)	28 (38.9)	8 (33.3)	20 (41.7)		184 (50.8)	
NICU admission	130 (30.0)	47 (65.3)	20 (83.3)	27 (56.3)	0.04	83 (22.9)	<0.001

Notes: median (interquartile range) or N (%); continuous variables compared using t-tests and Wilcoxon rank sum tests and categorical variables compared using Fisher’s exact tests

Abbreviations: GA = gestational age; NICU = neonatal intensive care unit

*p-value denotes significance between monochorionic versus dichorionic pregnancies

**p-value denotes significance between twin versus singleton pregnancies

### COVID-19 infection and vaccine history

Prior to delivery, whether prior to pregnancy or during pregnancy, 178, 154, and 66 participants received 2, 3, and 4 + COVID-19 vaccines, respectively. The number of COVID-19 vaccine doses prior to delivery or during pregnancy, median latency time between the last vaccine dose and delivery or gestational age at last vaccine dose were not significantly different among singleton and twin pregnancies (Table 1). No difference in the number of COVID-19 vaccines in pregnancy or median time between last vaccine dose and delivery was seen between mono- and dichorionic twin pregnancies, however individuals with dichorionic pregnancies were more likely to receive the last COVID-19 vaccine dose at an earlier gestational age than monochorionic pregnancies (p = 0.01). There was no difference in people with history of COVID-19 infection or positive anti-N serology between singleton and twin pregnancies or between mono- and dichorionic twin pregnancies (Table 1).

### Anti-S antibody analysis

**Fig 1:**
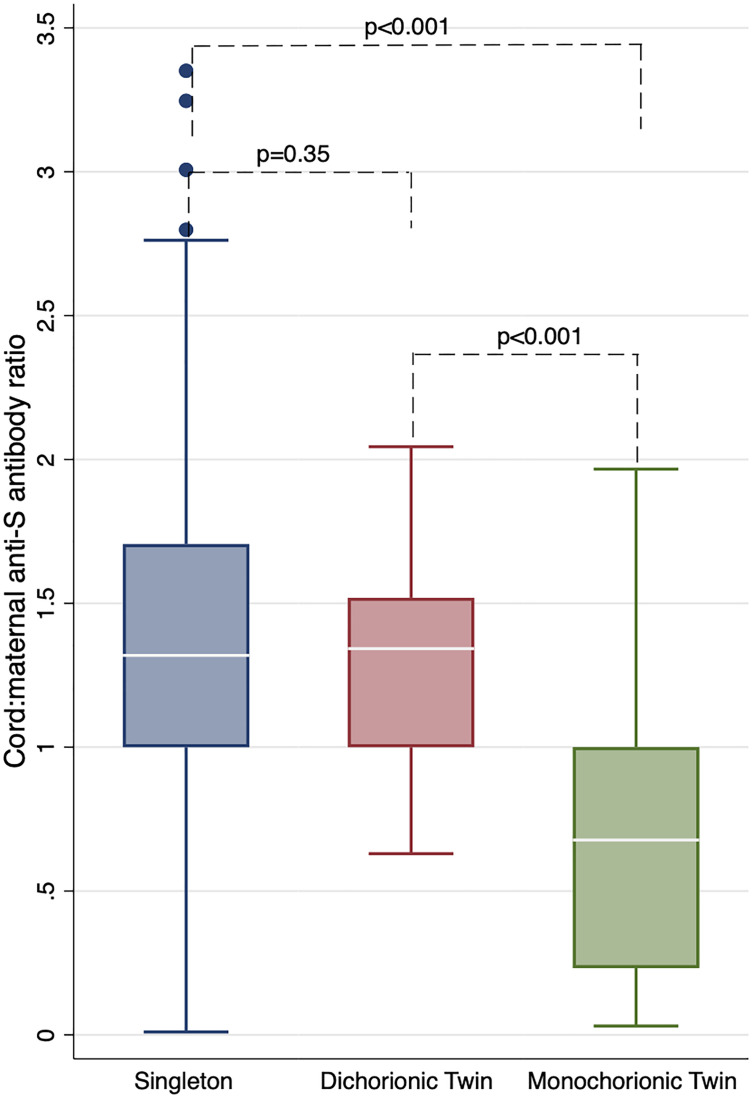
Boxplot of cord:maternal anti-Spike (S) antibody ratios in singleton and dichorionic and monochorionic twin pregnancies. Abbreviation: anti-S = anti-Spike antibody.

The unadjusted maternal anti-S antibody GMC was 3553 BAU/mL (95% CI 3017, 4185) and the unadjusted cord anti-S antibody GMC was 4083 BAU/mL (95% CI 3499, 4766) (Table 3). There was no difference seen between singleton and twin pregnancies (p = 0.96; p = 0.18) or between mono- and dichorionic twin pregnancies (p = 0.50; p = 0.21) when comparing the maternal and cord anti-S antibodies, respectively. The cord:maternal antibody ratio of singleton pregnancies was higher (1.23, 95% CI 1.17, 1.31) than twin pregnancies (0.87, 95% CI 0.69, 1.08; p < 0.001). Additionally, the cord:maternal antibody ratio of dichorionic pregnancies (1.25, 95% CI 1.14, 1.36) was higher than monochorionic pregnancies (0.42, 95% CI 0.24, 0.73; p < 0.001; Fig 1). We compared cord:maternal antibody ratio between singleton and dichorionic or monochorionic twin pregnancies. While there were no significant differences between transfer ratios in dichorionic twin pregnancies compared to singleton pregnancies (p = 0.35), cord:maternal antibody ratio of monochorionic pregnancies was lower than singleton pregnancies (p < 0.001).

**Table 3:** Maternal and cord anti-spike (S) antibody concentrations in singleton and twin pregnancies stratified by chorionicity.

	Total (n = 398)	Twin total (n = 36)				Singleton (n = 362)	p-value**
			Mono (n = 12)	Di (n = 24)	p-value*		
Maternal anti-S (BAU/mL)	3553 (3017, 4185)	3070 (1447, 6511)	4356 (1286, 14748)	2577 (944, 7035)	0.50	3605 (3057, 4253)	0.96
Cord spike anti-S (BAU/mL)	4083 (3499, 4766)	2657 (1538, 4591)	1821 (718, 4622)	3209 (1605, 6416)	0.21	4448 (3828, 5168)	0.18
Ratio: cord/maternal anti-S	1.16 (1.09, 1.24)	0.87 (0.69, 1.08)	0.42 (0.24, 0.73)	1.25 (1.14, 1.36)	<0.001	1.23 (1.17, 1.31)	<0.001

Notes: Geometric mean concentrations (95% confidence intervals); maternal and cord anti-spike IgG compared using Wilcoxon rank sum test; ratio is calculated as cord anti-spike IgG divided by maternal anti-spike IgG and compared using t-test; numbers for total and each group represent the number of pairs (total n for maternal ab = 398; total n for cord ab = 434; total n for ratio = 434)

Abbreviations: anti-S = anti-Spike antibody

*p-value denotes significance between monochorionic versus dichorionic pregnancies

**p-value denotes significance between twin versus singleton pregnancies

To test for the association between singleton and twin pregnancies and maternal and cord anti-S antibody levels we ran linear regression analysis and, in our fully adjusted model, we controlled for time between last vaccine dose and delivery, gestational age at delivery, fetal growth restriction and number of vaccine doses prior to delivery (Table 4). Both maternal and cord antibody concentrations were significantly lower in twin pregnancies compared to singleton pregnancies (b: −0.91, 95% CI −1.62, −0.19; p = 0.01; b:-1.20, 95% CI −2.36, −0.04; p = 0.04, respectively) although there was no difference in cord:maternal antibody ratios.

**Table 4:** Association between singleton/twin and maternal and cord anti-Spike (S) antibody, adjusting for covariates.

	β-coefficient (95% CI)	P-value	*Minimally Adjusted β-coefficient (95% CI)	P-value	^+^Fully Adjusted β-coefficient (95% CI)	P-value
**Maternal Anti-S** antibody						
Singleton	0.0 (ref)	–	0.0 (ref)	–	0.0 (ref)	–
Twin	−0.23 (−1.06, 0.59)	0.58	−0.77 (−1.61, 0.07)	0.07	−0.91 (−1.62, −0.19)	0.01
**Cord Anti-S** antibody						
Singleton	0.0 (ref)	–	0.0 (ref)	–	0.0 (ref)	–
Twin	−0.74 (−1.86, 0.37)	0.19	−1.02 (−2.19, 0.15)	0.09	−1.20 (−2.36, −0.04)	0.04
**Ratio: cord/maternal anti-S antibody**						
Singleton	0.0 (ref)	–	0.0 (ref)	–	0.0 (ref)	–
Twin	−0.27 (−0.44, −0.09)	0.003	−0.09 (−0.26, 0.08)	0.30	−0.08 (−0.26, 0.09)	0.36

*Adjusted for time between last dose and delivery in weeks and gestational age at delivery

+Adjusted for time between last dose and delivery in weeks, gestational age at delivery, fetal growth restriction, and number of vaccine doses prior to delivery

We ran a second adjusted linear regression analysis to test the association between chorionicity in twin pregnancies and maternal and cord anti-S antibody levels (Table 5). In this model we adjusted for timing of the last COVID-19 dose prior to delivery, gestational age at delivery and number of vaccine doses, and no difference was found for maternal or cord anti-S antibodies. However, cord:maternal antibody ratios were significantly lower in monochorionic pregnancies compared to dichorionic pregnancies (β: −0.49; 95% CI: −0.86, −0.13; p = 0.01).

**Table 5:** Association between monochorionic and dichorionic twin and maternal and cord anti-Spike (S) antibody, adjusting for covariates.

	β-coefficient (95% CI)	P-value	*Minimally Adjusted β-coefficient (95% CI)	P-value	^+^Fully Adjusted β-coefficient (95% CI)	P-value
**Maternal Anti-S antibody**						
Dichorionic	0.0 (ref)	–	0.0 (ref)	–	0.0 (ref)	–
Monochorionic	0.76 (−1.56, 3.08)	0.51	0.75 (−1.60, 3.11)	0.52	0.87 (−1.63, 3.37)	0.48
**Cord Anti-S antibody**						
Dichorionic	0.0 (ref)	–	0.0 (ref)	–	0.0 (ref)	–
Monochorionic	−0.82 (−3.17, 1.54)	0.49	−0.81 (−3.20, 1.59)	0.50	−0.60 (−2.92, 1.72)	0.60
**Ratio: cord/maternal anti-S antibody**						
Dichorionic	0.0 (ref)	–	0.0 (ref)	–	0.0 (ref)	–
Monochorionic	−0.57 (−0.93, −0.21)	0.003	−0.57 (−0.92, −0.21)	0.003	−0.49 (−0.86, −0.13)	0.01

*Adjusted for time between last dose and delivery in weeks

+Adjusted for time between last dose and delivery in weeks, number of vaccine doses prior to delivery and gestational age at delivery

## Discussion

Our study shows a significant difference in transplacental transfer of anti-S antibody between monochorionic and dichorionic or singleton pregnancies. Paired cord:maternal anti-S antibody ratios in dichorionic pregnancies resemble anti-S antibody ratios in singleton pregnancies, demonstrating efficient transplacental antibody transfer in both singleton and dichorionic pregnancies, similar to other published studies on SARS-CoV-2-specific antibody transfer ratios [23]. However, given significantly lower transplacental antibody transfer ratios in monochorionic pregnancies, an additional COVID-19 vaccine dose during pregnancy may help boost anti-S antibodies in order to optimize IgG levels available for transfer.

Our study focused on maternal antibody transfer post-COVID-19 vaccination in multiple gestation pregnancies and found that twins (both mono- and dichorionic pregnancies combined) has lower maternal and cord anti-S antibody levels compared to singleton pregnancies after covariate adjustment. Additionally, our data revealed significantly lower cord:maternal anti-Spike IgG ratios in mono- versus dichorionic twin pregnancies (p < 0.001). These findings are consistent with a prior study from 2014 investigating total IgG concentrations in mono- and dichorionic pregnancies by Stach et al. where monochorionic twins had lower total IgG transfer ratios than dichorionic twin infants [16]. However, in a further study from the same group specifically focused on umbilical cord serum concentrations and transfer ratios of IgG antibody directed against group B streptococcus, LPS from Klebsiella species and pseudomonas species, chorionicity was not associated with differences in IgG transfer ratios [24]. Lower cord:maternal antibody ratios can be potentially explained by multiple factors including gestational age at delivery (although we adjusted for this), and concentrations of FcRn receptors which increase in the 3^rd^ trimester. In addition, the nature of the antigen and specific antibody characteristics may affect antibody transfer [25–27]. Another proposed explanation is that transplacental transfer of IgG in monochorionic pregnancies are distributed between two fetuses via placental vascular anastomosis, leading to lower levels of IgG than if antibodies were distributed amongst two placentas [16,28]. While lower antibody ratios signify lower antibody transfer to the fetus relative to maternal antibody concentrations, they do not necessarily correlate with immune protection from a pathogen or correlates of protection which are often not known. Further research, specifically into placental factors involved in chorionicity and transplacental antibody transfer in twin pregnancies, may shed more light on our findings.

Current clinical guidelines for COVID-19 vaccine in pregnancy recommend at least one dose of an updated COVID-19 vaccine prior to delivery [29]. Optimization of maternal antibody levels prior to delivery appears to be advantageous for individuals with vaccine-mediated or hybrid immunity [30]. A growing proportion of the population has become infected with SARS-CoV-2, and the primary COVID-19 vaccine series is no longer available. Twin infants from both monochorionic as well as dichorionic pregnancies benefit from anti-S antibody transfer in potential protection against SARS-CoV-2 infection. In monochorionic pregnancies, an additional COVID-19 vaccine dose during pregnancy may help boost maternal anti-S antibodies in order to optimize transplacental antibody transfer to the fetuses. More studies are needed to elucidate whether additional vaccine boosters given to individuals with monochorionic pregnancies would boost anti-S antibody transfer to the significantly higher levels seen in singleton and dichorionic pregnancies.

As attention towards maternal immunization strategies grows, it is important to evaluate transplacental IgG transfer in the setting of high-risk pregnancy conditions such as monochorionic multiple pregnancies including investigations of the placenta and FcRn receptors. In addition, transplacental transfer relies on multiple factors including specific characteristics of the pathogen-specific antibody and the placenta. In the case of prior SARS-CoV-2 infection, studies have shown alterations to the placenta as well as potential augmentation of antibody transfer after COVID-19 infection [31,32]. Further studies into characteristics contributing to high-risk pregnancies may help optimize maternal immunization specifically for infants particularly at risk for infectious morbidity.

### Strengths and limitations

Our analysis encompasses a prospective cohort from an institution with large numbers of both low- and high-risk pregnancies, with relatively broad inclusion criteria, enabling us to increase generalizability of our results. Our methods to evaluate efficient transplacental transfer with cord:maternal anti-Spike antibody ratios at a threshold > 1 has been previously proposed in published literature [33]. We collected paired maternal and cord blood for all subjects with minimal missing variables which allowed for comprehensive assessment of pertinent outcomes related to maternal vaccination. In addition, the ability to link to the Washington State Vaccination Registry allowed abstraction of accurate vaccine data for our participants and is a strength of the study. Finally, the evaluation of COVID-19 vaccines and anti-S antibodies in multiple gestations with sub-analysis by chorionicity is a clinically important topic given the increased risk in these pregnancies for preterm delivery and low birth weight.

Although our study is unique in reporting on anti-S concentrations in pregnancies and multiple pregnancies stratified by chorionicity, we were limited by sample size, which comes with a few implications for the data. First, we found a significant difference between cord:maternal ratios, but not in maternal or cord antibody concentrations. With the small sample sizes, our ability to detect differences in maternal and cord concentrations may be limited. Second, fully-adjusted models may be overfitted and therefore, uncertainty remains around the results. Third, there may be additional confounders, measured and unmeasured, that we were not able to investigate due to the small sample size or unavailability of data. We do not report results from neutralizing assays, although previously published studies have shown good correlation with anti-S antibody levels and neutralizing function [34]. In terms of anti-S testing, the upper limit of Roche Elecsys® Anti-SARS-CoV-2 S immunoassay is 25,720 BAU/mL. Of our participants 64 (16.1%) of maternal samples and 70 (16.1%) of cord samples reached this level. It also tests predominantly for IgG but may pick up small quantities of IgM and IgA [35]. While these immunoassay characteristics do not affect maternal and cord antibody concentration analyses, there may be limitations for interpretation of cord:maternal antibody ratios.

## Conclusions

The results of the study suggest that chorionicity in twin pregnancies and not multiple gestation itself affects the transfer of maternal anti-S IgG transfer in pregnancy. Although pregnancies included in our study displayed significant transplacental antibody transfer following vaccination with two or more doses of an mRNA COVID-19 vaccine regardless of number of fetuses or placentas, monochorionic twin pregnancies showed reduced antibody transfer ratios compared to dichorionic twin pregnancies and singleton pregnancies. Maternal immunization with the COVID-19 vaccine in twin pregnancies and particularly in monochorionic pregnancies to augment maternal IgG antibody concentrations and thus cord antibody concentrations may be beneficial for improving infant immune protection against COVID-19.
